# Novel transcriptional profile in wrist muscles from cerebral palsy patients

**DOI:** 10.1186/1755-8794-2-44

**Published:** 2009-07-14

**Authors:** Lucas R Smith, Eva Pontén, Yvette Hedström, Samuel R Ward, Henry G Chambers, Shankar Subramaniam, Richard L Lieber

**Affiliations:** 1 Department of Bioengineering, University of California San Diego, La Jolla, California, USA; 2 Dept of Woman and Child Health, Pediatric Orthopedic Surgery, Karolinska Institute, Stockholm, Sweden; 3 Department of Clinical Neurophysiology, Uppsala University, Uppsala, Sweden; 4 Department of Radiology, University of California San Diego, La Jolla, California, USA; 5 Rady Children's Hospital San Diego, San Diego, California, USA; 6 Department of Orthopaedic Surgery, University of California San Diego, La Jolla, California, USA

## Abstract

**Background:**

Cerebral palsy (CP) is an upper motor neuron disease that results in a progressive movement disorder. Secondary to the neurological insult, muscles from CP patients often become spastic. Spastic muscle is characterized by an increased resistance to stretch, but often develops the further complication of contracture which represents a prominent disability in children with CP. This study's purpose is to characterize alterations of spastic muscle on the transcriptional level. Increased knowledge of spastic muscle may lead to novel therapies to improve the quality of life for children with CP.

**Method:**

The transcriptional profile of spastic muscles were defined in children with cerebral palsy and compared to control patients using Affymetrix U133A chips. Expression data were verified using quantitative-PCR (QPCR) and validated with SDS-PAGE for select genes. Significant genes were determined using a 2 × 2 ANOVA and results required congruence between 3 preprocessing algorithms.

**Results:**

CP patients clustered independently and 205 genes were significantly altered, covering a range of cellular processes. Placing gene expression in the context of physiological pathways, the results demonstrated that spastic muscle in CP adapts transcriptionally by altering extracellular matrix, fiber type, and myogenic potential. Extracellular matrix adaptations occur primarily in the basal lamina although there is increase in fibrillar collagen components. Fiber type is predominately fast compared to normal muscle as evidenced by contractile gene isoforms and decrease in oxidative metabolic gene transcription, despite a paradoxical increased transcription of slow fiber pathway genes. We also found competing pathways of fiber hypertrophy with an increase in the anabolic IGF1 gene in parallel with a paradoxical increase in myostatin, a gene responsible for stopping muscle growth. We found evidence that excitation-contraction coupling genes are altered in muscles from patients with CP and may be a significant component of disease.

**Conclusion:**

This is the first transcriptional profile performed on spastic muscle of CP patients and these adaptations were not characteristic of those observed in other disease states such as Duchenne muscular dystrophy and immobilization-induced muscle atrophy. Further research is required to understand the mechanism of muscle adaptation to this upper motor neuron lesion that could lead to the development of innovative therapies.

## Background

Cerebral palsy (CP) is a disorder in which children experience a non-progressive brain lesion that results in permanent and progressive secondary postural and movement disorders [1]. CP has an incidence of 2.0–2.5 occurrences per 1000 live births in developed nations, making it the most common cause of physical disability in children [2]. There is a spectrum of disease states in CP that affect upper and lower limbs to varying degrees. Since the primary lesion in CP is in the central nervous system, most CP research has been focused on the neurological disorder [3-5]. However, since the secondary effects of CP disrupt posture and movement, most conservative and surgical treatments address the musculoskeletal system [6].

It is clear that skeletal muscles from CP patients are altered secondary to the neurological lesion. There are many neurological symptoms secondary to the brain lesion including dystonia, ataxia, athetosis and particularly spasticity [7,8]. Loss of upper motor neuron (UMN) inhibition on the lower motor neurons (LMN) results in spasticity, altered muscle tone and increased/impaired motor unit firing. Loss of UMN excitation of LMNs leads to negative features of UMN syndrome that include weakness, fatigability, poor balance, and occasionally, sensory deficits. Although the mechanism is unknown, spastic muscle often shortens to create muscle contractures, which is a primary disability of CP that leads to further complications [6]. There are many clinical approaches to managing spasticity to increase function, potentially decrease muscle contractures and most importantly improve quality of life. Oral medications, physical therapy techniques, chemical neurectomies with phenol or alcohol, chemodenervation using neurotoxins (BTX), and surgical neurectomies have all been utilized to decrease spasticity in children with CP [9]. Unfortunately, while there has been some success in this management, many children ultimately require orthopedic surgery to lengthen the tendons of contracted muscle so that arm or leg function can be increased. If the adaptation of the muscle tissue were more completely understood, it might lead to novel medical treatments of contractures.

Skeletal muscle from children with CP has been characterized at a variety of levels, with most studies reporting muscle tissue and muscle fiber atrophy, decreased muscle cross-sectional area, muscle shortening, and decreased specific tension [10,11]. All of these changes implicate physiological mechanisms of growth being involved in the pathology of muscle from CP patients. Interestingly, recent intraoperative studies of human muscles revealed abnormally long muscle sarcomere lengths in vivo [12] that were associated with muscle tissue of altered properties. Specifically, muscle fiber sarcomere length under no load (i.e., slack sarcomere length) was significantly decreased while the muscle tissue itself contained a hypertrophic extracellular matrix of poor material quality [11,13]. These changes implicate the mechanical force generating system of the muscle cell as well its extracellular matrix tissue. Muscle has been shown to adapt its mechanical function to neurological input [14], however the mechanism by which UMN lesion could lead to alterations in muscle myogenesis, force generation, force transmission and extracellular matrix properties is unknown. While there is evidence that neurotrophic factors dramatically affect muscle properties [15,16], there is neither mechanistic understanding as to how such factors might alter tissue properties, nor information as to which specific biosynthetic pathways might lead to these changes.

To develop an understanding of the physiological processes altered in spastic muscle secondary to CP, we exploited the fact that muscle tissue from a previous study, in which the clinical severity of the spasticity was clearly established, was available for transcriptional profiling [17]. We used GeneChip technology to contrast the transcriptome from CP patients with age-matched control patients whose muscles were completely normal. We performed a variety of analyses to identify a robust set of genes that were significantly altered in CP and interpreted these genes in their biological context to explain previously defined muscle changes. We also compared our transcriptional data to two other disease states to determine whether spasticity secondary to CP results in a unique muscle disorder at the gene expression level.

## Methods

### Muscle Sampling

Children were recruited for this study because they were receiving tendon transfers of the flexor carpi ulnaris (FCU) muscle into the extensor carpi radialis brevis (ECRB), the extensor carpi radialis longus muscle, or the extensor digitorum communis muscles [18]. All patients had CP and developed a contracture indicating surgery, despite receiving conservative treatment that included splinting and occupational therapy. Parental consent and child's assent was obtained in accordance with our institutional review boards. From the original sample size of 23 [18], a subset (n = 6 children, average age 12.8 ± 1.5 years) was selected to cover a range of clinical severities determined from the House [19], Ashworth [20], and Zancolli [21] classification systems as well as characteristics of sarcomere length and range of motion. Control tissue was obtained incidentally (n = 2 children, average age 8.5 ± 2.1 years) from both the FCU and ECRB muscles in children with no previous history of any neural injury who were undergoing surgery for forearm fracture repair. None of the surgeries injured the control muscles in any way. We suggest that these samples represent true muscle controls for the following reasons: 1) the surgeon verified that the muscles from which biopsies were taken were in pristine condition and showed no signs of damage, 2) surgery was emergent in these children, and therefore, control samples were obtained within 24 hours of fracture, 3) controls showed no significant effect for many of the transcripts associated with trauma or immobilization and were, in fact, often altered in the opposite direction (data not shown) (21, 60). Just prior to harvesting of the spastic muscle biopsies, sarcomere length of the FCU was measured by laser diffraction *in vivo*. While the wrist was held in neutral, a small fiber bundle was transilluminated with a HeNe light. The sarcomere length could be calculated from the diffraction pattern obtained [18]. CP biopsies were snap frozen in isopentane chilled by liquid nitrogen (-159°C), and stored at -80°C until analyzed (Table 1). No patient had undergone serial casting prior to surgery, two patients (AN and BF) had BTX injections into the FCU several months prior to surgery, and one patient (AQ) had a prior BTX injection in the biceps.

**Table 1:** Primers used for quantitative PCR

**Gene**	**Transcript**	**Base pairs**	**Sense Primer (5'-3')**	**Antisense Primer (5'-3')**
*COL1A2*	NM_000089	225	TCCAAAGGAGAGAGCGGTAA	GCCACTTGCACCACGACTA
*COL4A2*	NM_001846	269	CTGGGTGGCGGAGTTTGTG	GCTGATGTGTGTGCGGATGAG
*COL4A3*	NM_000091	157	CACCAGCTCTGATGCCAATG	AGAGAAATCCAGCCGTGAGG
*DMD*	NM_004019	151	GACCAGCACAACCTCAAGCAA	TCAGCAGCCAGTTCAGACACA
*FBXO32*	NM_058229	376	GTCCAAAGAGTCGGCAAGT	TTGGGTAACATCGGACAAGT
*GAPDH*	NM_002046	172	TCTGACTTCAACAGCGACAC	TGGTCCAGGGGTCTTACTC
*IGF1*	NM_000618	355	AGCAGTCTTCCAACCCAATTA	CACGGACAGAGCGAGCTG
*IGFBP5*	NM_000599	339	CCAAACACACCCGCATCT	CAGCTTCATCCCGTACTTGTC
*GDF8*	NM_005259	167	TATCACGCTACAACGGAAAC	GGAGTCTCGACGGGTCTC
*MYH1*	NM_005963	132	AAGAGCAGGGAGGTTCACAC	TTATCTCCAAAAGTCATAAGTACA
*NEB*	NM_004543	131	CCGTGCCATGTATGACTATAT	CGGTCCTGCCAGTCCTCTG
*PVALB*	NM_002854	329	GATGACAGACTTGCTGAACGC	CTTAGCTTTCAGCCACCAGAG
*TRIM63*	NM_032588	393	GAGGATTCCCGTCGAGTGAC	AATGGCTCTCAGGGCGTCT

Table of primers used for QPCR analysis. Genbank accession number and PCR product length given in parentheses after each transcript name

### RNA preparation and gene expression profiling

RNA was extracted using a combination of standard Trizol (Invitrogen, Carlsbad, CA) and RNeasy (Qiagen, Valencia, CA) protocols. Briefly, 30 mg of frozen muscle was homogenized in a rotor-stator homogenizer on ice in 0.5 ml of Trizol; 0.1 ml of chloroform was added to the solution, which was then vigorously vortexed for 15 s followed by centrifugation at 4°C for 15 min. The upper aqueous layer was removed and mixed with an equal volume of 70% ethanol before being added to the RNeasy spin column. After the column was washed, it was incubated with RNAse-free DNAse (Qiagen) for 15 min and then washed again three more times before being eluted as described in the manufacturer's protocol. RNA concentration was determined by the absorbance at 260 nm, and the 260 nm-to-280 nm absorbance ratio was calculated to define RNA purity.

### Microarray data analysis

Affymetrix microarrays ("GeneChip" HG-U133A; Affymetrix, Santa Clara, CA) were used for each muscle biopsy (n = 16 chips; 2 muscles × 8 patients) and the data are available [GEO: GSE11686]. RNA processing for the GeneChip, including stringent quality control measures, was performed by the Gene Chip Core at the Department of Veterans Affairs San Diego Health Care System, (San Diego, CA). GeneSpring software (version 7.3; SilconGenetics, Redwood City, CA) was used to identify those genes that were significantly altered in CP. Initially, a 12.5% (2/16 chips) present call on MAS5 (Affymetrix) was used to filter out poorly performing probe sets in the analyses. Three independent probe set algorithms were used for signal generation and normalization: MAS5, RMA, and GCRMA. Recent reports support requiring concordance among different probe set algorithms as an approach to reduce false positives in data sets [22-24]. Each feature was normalized per chip (to the median of all features on each chip) and per gene (to the median of that feature on all chips). Normalized gene values were subjected to a 2 × 2 Welch ANOVA of muscle type (FCU vs. ECRB) and disease state (CP vs. CTRL) with a required statistical significance (P < 0.05) with a Benjamini and Hochberg False Discovery Rate (FDR) multiple testing correction for present features. Thus 5% of the genes deemed significant for an individual preprocessing algorithm are suspected to be false positives. Features that passed in all three preprocessing algorithms were deemed significantly altered in CP.

The condition tree was created using a Pearson Correlation similarity score and average linkage clustering algorithm for all samples on present features. For severity analysis, a Welch ANOVA for each severity parameter was run on the MAS5 data of the features deemed significantly altered in CP without the control patients and a required statistical significance of (P < 0.05) also with an FDR multiple testing correction.

Promoter sequence analysis was conducted using GeneSpring on the list of genes altered in CP. The upstream sequence from -10 to -1000 base pairs was analyzed for a nucleotide sequence of from 6 to 10 nucleotides long and containing at most 2 N values in the middle. Significance was determined based on the number of times the given sequence appeared in the upstream sequence of all other genes and was corrected for multiple testing. The analysis was performed on the whole list of genes altered in CP and the sub lists of up- or down-regulated.

### Quantitative real-time PCR

QPCR was performed to validate expression levels of selected genes to the GeneChip data and to provide mRNA expression levels for genes not contained on the HG-U133A chip. After RNA was extracted from the muscle as described previously and diluted 1:5 with DNase/RNase free water (Invitrogen), 1 μl of each sample was reverse transcribed using standard protocols (Superscript III; Invitrogen). cDNA was amplified with the Cepheid SmartCycler (Sunnyvale, CA) with primers specific to the genes of interest (Table 2). All primers were tested for cross-reactivity with other transcripts using nBLAST and Oligo (version 6.6; Molecular Biology Insights, Cascade, CO). All samples were run at least in triplicate, along with a standard curve. The PCR reaction vessel (25 μl) contained 1× PCR buffer, 2 mM MgCl_2 _(Invitrogen), 0.2 mM sense and antisense primers, 0.2 mM dNTP, 0.2× SYBRgreen, and 1 U of platinum Taq polymerase (Invitrogen). Amplification conditions were as follows: An initial hold at 95°C for 2 min was followed by 40 cycles of denaturing at 95°C for 15 s, followed by annealing/extension at 68°C for 40 s. The success of each reaction was deduced based on the observation of a single reaction product on an agarose gel and a single peak on the DNA melting temperature curve determined at the end of the reaction. To express QPCR results, we used the standard curve method with the "cycles to threshold" value representing the number of PCR cycles at which the SYBRgreen signal was increased above the threshold. Each sample's value was measured in triplicate, normalized to the housekeeping gene GAPDH, and then averaged. QPCR data were normalized to the median value of the gene to permit comparison to the GeneChip data.

**Table 2:** Subject information and clinical evaluations

**Sub**	**Sex**	**Age**	**Muscle**	**SL (μm)**	**House**	**Sev**	**Ash**	**Zan**	**PEFF (°)**	**PESF (°)**	**AEFF (°)**	**MyHC gel**
*AI*	M	13	FCU	6.36	1	SS	4	3	-5	-90	-70	x
			ECRB									x
*AN*	F	12	FCU	4.56	3	SS	3	2B	50	45	-90	
			ECRB									
*AO*	F	11	FCU	4.01	5	SM	2	2B	65	60	-50	x
			ECRB									x
*AQ*	M	15	FCU	4.01	7	M	2	2B	80	80	-50	
			ECRB									
*AT*	M	14	FCU	3.57	0	SS	3	3	45	40		
			ECRB									
*BF*	F	12	FCU	5.31	6	SM	3	2A	85	85	55	x
			ECRB									x
*BP*	M	10	FCU	-	-	C	1	0	90	90	90	x
			ECRB									
*AZ*	M	7	FCU	-	-	C	1	0	90	90	90	x
			ECRB									x

Table of the eight subjects used in this study with 2 muscles (FCU and ECRB) per patient with sex and age recorded. Measures of severity were taken to include: intraoperative measurement of sarcomere length (SL) on the FCU, House clinical assessment of activity, Severity (Sev) grouped by House (Severe Severe (SS), Severe Moderate (SM), Mild (M), and Control (C)), Ashworth (Ash) clinical assessment, Zancolli (Zan) classification based on finger extension, passive extension with flexed fingers (PEFF) passive extension with straight fingers (PESF) and active extension with flexed fingers (AEFF). Samples that were available for MyHC SDS-PAGE gels are noted in the final column

### Myosin protein content biochemistry

Myosin heavy chain protein content was measured (Table 2) for comparison to the GeneChip data as previously described [25]. Three bands were identified corresponding to MyHC I, MyHC IIa/fetal and MyHC IIx/embryonic. (Using this methodology, embryonic MyHC cannot be separated from MyHC IIx and fetal MyHC cannot be separated from MyHC IIa.) The gels were scanned in a soft laser densitometer (Molecular Dynamics Sunnyvale, CA, USA). The relative proportion of each MyHC isoform was determined by using a densitometric system (ImageQuant TL Software v 2003.01, Amersham Biosciences, Uppsala, Sweden).

### Gene Ontology analyses

Gene ontology analysis provides a means of converting a list of differentially expressed genes into a hierarchical list of gene ontologies that are significantly altered. We used the web-based software GOTree ; [26]) to compare the list of features altered in CP to the list of features present on the HG-U133A chip. In this analysis, a P-value is generated for each ontology based on hyperbolic comparison of the number of genes present in that list to the number of genes expected to be present based on the size of the list. The analysis was performed on the entire list of genes altered in CP, with a required P < 0.01.

### Biological pathway analyses

To gain understanding into the biological context of transcript changes, we investigated the way in which genes were involved in various muscle pathways. We analyzed pathways from databases including: Ingenuity Pathway Analysis (IPA; ), Kyoto Encyclopedia of Genes and Genomes (KEGG; ), and Gene Map and Pathway Profiler (GenMAPP; ). These pathways permit establishment of pathways specific to muscle involving critical muscle functions such as: neuromuscular junction function, excitation-contraction coupling, muscle contraction, extracellular matrix formation, muscle hypertrophy/atrophy, myogenesis, and fiber type switching. Based on the pathway databases and relying heavily our own literature review, we created pathways specific to muscle with particular emphasis on genes altered in CP. To quantify gene expression for pathway analysis, MAS5 data were normalized to the averaged control data within each muscle type and across CP samples. This value is termed the expression ratio.

Finally, to compare the CP transcriptome to other conditions we examined the pathways specific to muscle against transcriptome deposited for three other disease states, Duchenne Muscular Dystrophy (DMD; GSE465; [27]), immobilization (IMB; GSE8872; [28]), and hereditary spastic paraplegia (HSP; GSE1300; [29]). The DMD experiment used muscle from patients age 6–9 years and further details are described in the reference [27], but we compared data only from those U95A chipset. The IMB experiment used medial gastrocniemus muscle from adult patients and further details are described in the reference [28], but we compared only data from voluntary controls and ankle facture patients immobilized for 4–9 days. The HSP experiment used vastus lateralis muscle from adult patients and further details are described in the reference [29], but we used the U133A chipset and controls (1–10) from GSE3307. As these data sets are from subjects of different ages and muscles, and are acute (in the case of 4–9 days of immobilization), direct comparison to our CP dataset is somewhat problematic; however, we are able to investigate whether similar transcriptional trends are present for these muscle conditions. The expression ratio for each feature was taken as the MAS5 ratio of the average disease state:average control state of the particular study so disease values are normalized to their own controls. The genes expression ratio of a pathway for each disease (CP, DMD, IMB, HSP) was log averaged across the pathway with inverse expression values used for inhibitors. We similarly investigated a list of genes involved in satellite cell states of quiescence and activation [30-32].

## Results

Of the 22,283 probe sets on the HG-U133A GeneChip, 11,312 met the criteria of being "present" on 2/16 GeneChips and were therefore considered for further analysis. The number of genes that were significant for CP (P < 0.05) on the 2 × 2 Welch ANOVA of disease state and muscle (CP vs. CTRL; flexor carpi ulnaris (FCU) vs. extensor carpi radialis brevis (ECRB)) with FDR among the three preprocessing algorithms were: 495 for Microarray Suite Version 5.0 (MAS5), 1,141 for Robust Multiarray Analysis (RMA), and 1,207 for GCRMA. The overlap of these 3 preprocessing algorithms produced a final list of 205 genes (319 features) that were considered significantly altered secondary to CP (Sup. Table 1). Of these, more were up-regulated (143 genes, 220 features) than down-regulated (62 genes, 99 features). Table 3 reports the 72 genes subset of these 205 genes that were considered relevant to specific muscle functions. Genes in the Tables (Table 1; Sup. Table 1) are reported with the P-value for each preprocessing algorithm as well as the expression ratio. The 2 × 2 ANOVA yielded no genes significant (P < 0.05) for muscle type and only one gene with a significant interaction, MYH1. This was due to control ECRB tissue having a very low MYH1 mRNA content. This important result supports our previous contention that, even though these children present with wrist flexion contracture, the FCU and ECRB are equally affected and the wrist flexion simply results from the large size of the FCU [18].

**Table 3:** Significantly altered genes in functional categories

		**P-Values**					**P-Values**		
**GENE**	**Ratio**	**MAS5**	**RMA**	**GCRMA**	**GENE**	**Ratio**	**MAS5**	**RMA**	**GCRMA**
Neuromuscular Junction					Muscle Contraction and Structure				

*KCNN3*	12.98	0.013	0.011	0.018	*MYH1*	8.57	0.001	0.001	0.000
*COL4A3*	2.89	0.043	0.014	0.019	*MYH4*	4.33	0.001	0.002	0.015
*LAMB2*	1.74	0.042	0.018	0.015	*NEB*	2.54	0.015	0.008	0.013
					
Excitation Contraction Coupling					*MYBPC2*	2.16	0.027	0.004	0.004
					
*PVALB*	62.60	0.001	0.004	0.001	*DMD*	2.11	0.023	0.020	0.027
*ATP2B2*	2.60	0.033	0.023	0.021	*LDB3*	1.93	0.030	0.015	0.015
					
*TRDN*	2.38	0.028	0.019	0.017	Metabolism/Mitochondria Related				
					
*ATP2C1*	2.30	0.046	0.047	0.041	*WARS*	0.64	0.044	0.040	0.019
*PDE4DIP*	2.10	0.005	0.003	0.004	*CAV1*	0.64	0.037	0.013	0.017
*CALM1*	1.70	0.005	0.004	0.008	*CERK*	0.63	0.033	0.006	0.014
*CACNB1*	1.51	0.045	0.014	0.017	*A2M*	0.62	0.015	0.016	0.010
*FKBP1A*	0.56	0.039	0.016	0.011	*MDH1*	0.60	0.050	0.017	0.011
					
Myogenesis/Fiber type pathways					*PECI*	0.58	0.029	0.024	0.012
					
*GDF8*	3.65	0.025	0.032	0.007	*MRPL35*	0.57	0.040	0.043	0.037
*IGF1*	2.63	0.013	0.010	0.008	*MRPS18B*	0.56	0.009	0.002	0.001
*IGFBP5*	2.48	0.002	0.000	0.000	*SLC25A20*	0.55	0.044	0.049	0.022
*PLCB1*	2.34	0.028	0.014	0.007	*CPT2*	0.54	0.044	0.022	0.013
*RASA4*	2.26	0.037	0.027	0.029	*MRPS12*	0.53	0.044	0.008	0.010
*PPP3CA*	1.95	0.012	0.003	0.005	*UCP2*	0.51	0.030	0.023	0.015
*PBX1*	1.86	0.028	0.010	0.007	*MLYCD*	0.48	0.017	0.004	0.001
*CALM1*	1.70	0.005	0.004	0.008	*PPIF*	0.48	0.014	0.011	0.010
*MBNL1*	1.55	0.033	0.015	0.024	*ADM*	0.47	0.019	0.012	0.018
*MEF2A*	1.53	0.028	0.006	0.007	*UCP3*	0.47	0.014	0.010	0.007
*NEO1*	1.44	0.015	0.005	0.001	*ALDH6A1*	0.46	0.023	0.013	0.007
*HMGB1*	1.34	0.015	0.013	0.015	*ACSL1*	0.43	0.014	0.008	0.004
					
Extracellular Matrix					*GOT1*	0.42	0.039	0.027	0.021
					
*MFAP5*	3.64	0.012	0.004	0.014	*TST*	0.41	0.015	0.002	0.001
*COL4A3*	2.89	0.043	0.014	0.019	*MT1G*	0.37	0.015	0.009	0.011
*COL21A1*	2.86	0.021	0.022	0.015	*LPL*	0.35	0.013	0.010	0.007
*KAL1*	2.57	0.002	0.003	0.003	*RETSAT*	0.35	0.002	0.006	0.005
*MATN2*	2.24	0.013	0.011	0.007	*MT1M*	0.34	0.015	0.006	0.004
*CILP*	2.09	0.035	0.043	0.034	*MT1X*	0.32	0.014	0.012	0.010
*SMC3*	1.88	0.021	0.026	0.019	*GLUL*	0.31	0.018	0.017	0.013
*ECM2*	1.86	0.037	0.035	0.021	*MT2A*	0.29	0.015	0.013	0.011
*LAMB2*	1.74	0.042	0.018	0.015	*MT1H*	0.29	0.015	0.015	0.012
*COL4A1*	0.51	0.048	0.032	0.015	*MT1F*	0.27	0.019	0.012	0.009
*BSG*	0.48	0.033	0.026	0.030	*MT1E*	0.26	0.012	0.006	0.005
*COL4A2*	0.43	0.012	0.010	0.006	*LIPE*	0.22	0.028	0.001	0.002

Table of genes within function groups related to skeletal muscle. Groups defined by ratio is the expression ratio of CP:CTRL. P-values are listed for the three separate preprocessing algorithms used (MAS5, RMA, GCRMA).

Promoter sequence analysis performed on each gene altered in CP did not reveal any 6–10 base pair sequences that were overrepresented 10–1000 base pairs upstream of the gene. Of course, regulation can occur farther upstream than 1000 base pairs and regulation sequences can be outside of the 6–10 base pair range. Thus, further sequence analysis may reveal significant promoter or enhancer sequences, but none were identified using these criteria.

### Condition tree correlates with clinical severity scores and treatment

**Figure 1:**
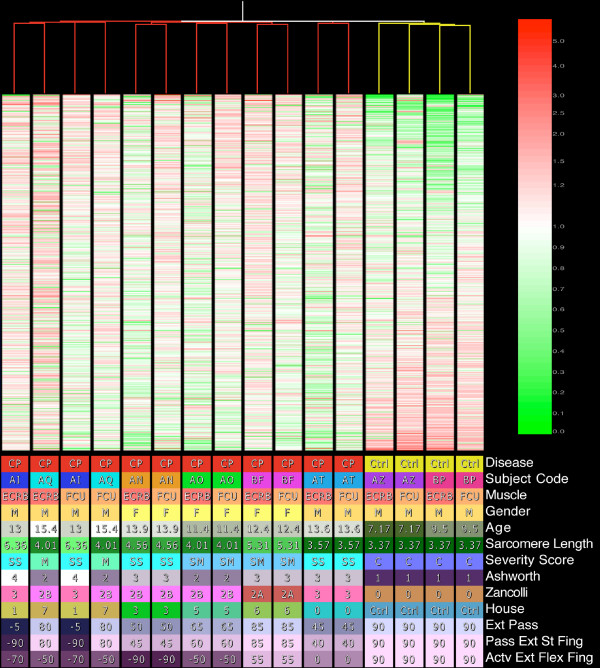
**Top: Condition tree created using Pearson Correlation for the similarity score and an average linkage clustering algorithm**. The tree was created based on all present features. MAS5 data were used with expression values normalized to each features median. Features are ordered from highest expression ratio to lowest. Bottom: Clinical conditions color-coded with values for each sample.

The condition tree resulted in the control patients being clustered together separate from CP patients (Figure 1). Figure 1 illustrates the condition tree based on all present genes. The tree shows that patients are grouped together in most cases rather than by muscle type suggesting more between-patient than between-muscle variability.

We had hoped that clinical severity [1,19-21] would allow us to define transcriptionally, the severity of CP or differences between flexor and extensor muscles. In this way, clinical parameters would be seen as representative of the state of the muscle tissue. These parameters were applied to an analysis of all of the 319 features altered in CP (Additional File 1 Table S1) but only sarcomere length and active wrist extension with fingers flexed had features that were significant, containing the same two genes, *RBM9 *and *RHOBTB1*. Heat plot of these data reveals that even these genes undergo a much larger change in expression from the control sarcomere length (3.37 μm) to CP sarcomere lengths than they do in CP progression (data not shown). Thus it appears that our study is underpowered to reveal transcriptional correlation with clinical severity scores.

Treatment with BTX was investigated by comparing injected muscles vs. non-injected CP muscles, muscle from patients receiving injection of any muscle vs. non-injected patient muscle, and injected FCU muscles vs. non-injected FCU muscles. None of these analyses yielded any genes that met our requirement for statistical significance, and thus we show no significant transcriptional effect of BTX injection.

### RT-PCR results compared to chip results

**Figure 2:**
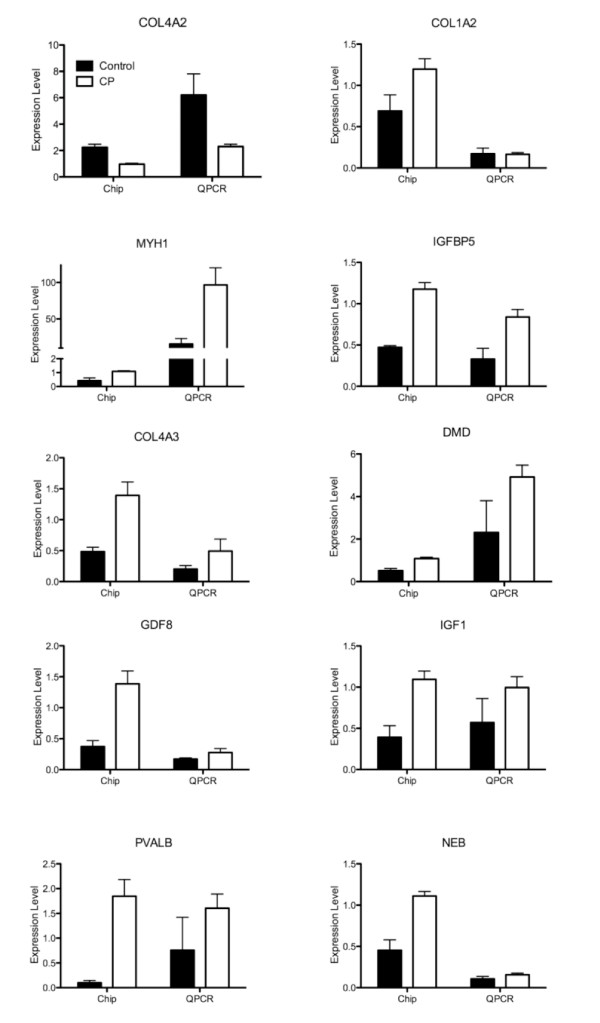
**QPCR results compared to GeneChip results for several individual genes shown from CP (open bars) and CTRL (filled bars) patients**. Error bars represent SEM. QPCR data are from dilute (1:100) samples to test multiple genes and represent transcript level relative to total RNA (fg/μg). GeneChip data are normalized to the median value for each gene and averaged across CP or CTRL samples.

**Figure 3:**
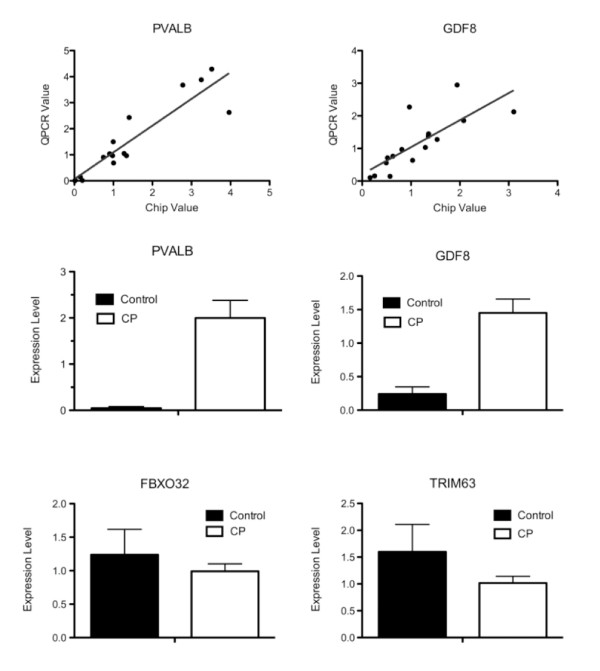
**(A/B) Sample-to-sample correlation between the QPCR and GeneChip results**. (A) PVALB, parvalbumin; (B) GDF8, myostatin. The solid line is a best fit regression line. (C-F) QPCR results showing the difference between CP and CTRL patients for specific genes, (C) PVALB, (D) GDF8, (E) FBXO32/MaFBX/Atrogin, (F) TRIM63/MuRF. Values are expressed determined relative to GAPDH and normalized to the median value for each individual gene. (*) represents significant difference (P < 0.05). Error bars represent SEM.

As a quality control measure, correlation of data between the GeneChip and QPCR was highly significant (P < 0.001) indicating internal consistency. To validate the GeneChip data, 10 genes covering a variety of cellular processes and expression levels were compared directly to transcript levels determined by QPCR on the same cDNA samples (Figure 2). For 9/10 genes studied, the direction of the transcript change (i.e., up- or down-regulation) was confirmed, and there was a good correlation between methodologies in terms of the magnitude of the effect. For two genes, (*PVALB*, *GDF8*) expression levels were evaluated relative to *GAPDH *transcript levels. These genes were selected based on their significant differences on the chip and their relevance to the disease state. Significant positive correlations were observed for both genes (*PVALB*, r^2 ^= 0.924, P < 0.001; *GDF8*, r^2 ^= 0.864, P < 0.001). The QPCR data were also subjected to 2 × 2 ANOVA (CP vs. CTRL; FCU vs. ECRB) and both *PVALB *and *GDF8 *were confirmed as significantly up-regulated in CP (Figures 3C and 3D).

Two genes of particular interest that are related to muscle atrophy, MAFbx (*FBXO32*) and MURF1 (*TRIM63*) were not represented on the chip [28,33]. Their expression was determined in the same manner as the genes described above (Figures 3E and 3F). Both of these genes were down-regulated in CP, but neither reached statistical significance.

### Myosin heavy chain protein-mRNA comparison

**Figure 4:**
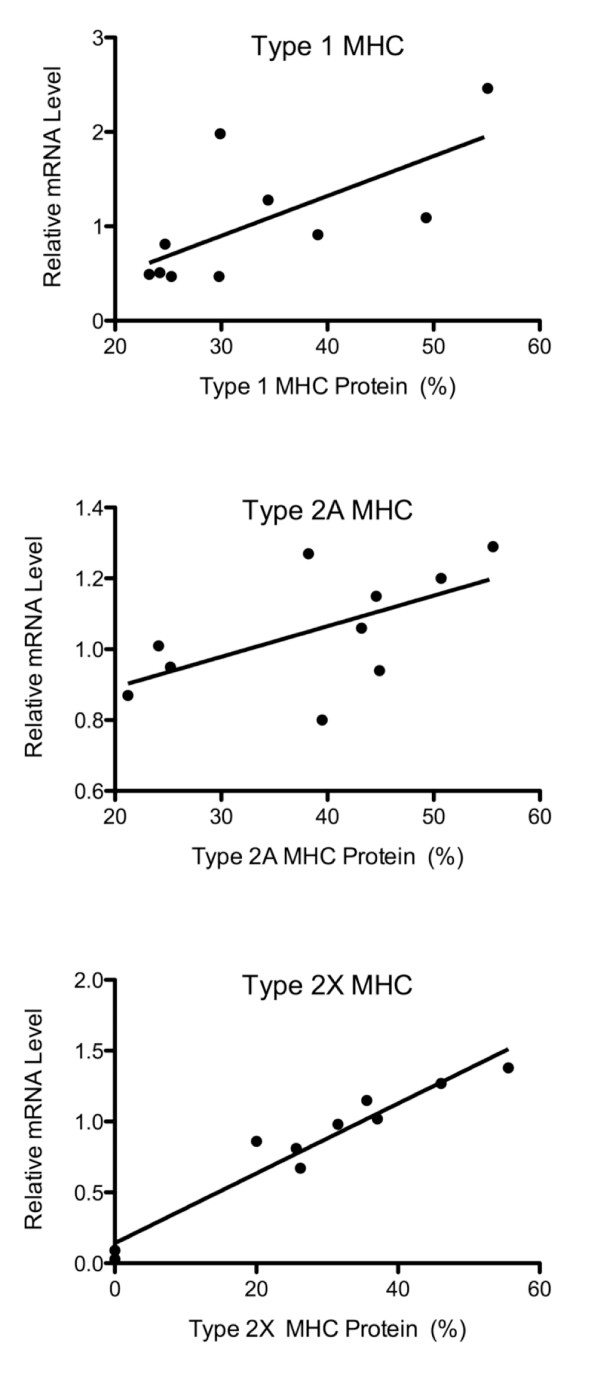
**Comparison of the GeneChip mRNA data to protein SDS-PAGE gel data for three myosin heavy chains commonly expressed in human skeletal muscle**. A: Type 1 MHC (MYH6), B: Type 2A MHC (MYH2), C: Type 2X MHC (MYH1). mRNA data are normalized to the median value for each gene on the chip using MAS5 preprocessing and protein data are normalized to total MyHC content.

The GeneChip and QPCR provide only transcriptional data and we wished to determine whether the transcriptional changes resulted in translational changes in the case of myosin heavy chain (MHC) for these samples [25,34]. All of the muscles were of a mixed fiber type, however the control ECRB tissue showed no evidence of type 2X MHC. The spastic muscles had a higher proportion of fast fibers than the controls of the corresponding muscle, with most of the increase in type 2X MHC. Comparison between protein and mRNA was confounded by the fact that MHC expression was normalized as percent of total myosin while mRNA was normalized to the median of that individual transcript across subjects. In spite of this difference, we still expected to see the same trend across samples, which was the case for type 1 MHC (gene *MYH8*) and type 2X MHC (gene *MYH1*) where protein and mRNA levels were significantly correlated (Figures 4A and 4C; P < 0.05) while type 2A MHC (gene *MYH4*) did not quite reach significance (Figure 4B; P = 0.065). Taken as a whole, these results suggest that, in the case of the MHC, protein levels reflected transcript levels.

### Gene ontology analysis

Thirty-eight different ontologies were overrepresented based on the 143 up-regulated genes (Additional File 2 Table S2; Additional File 3 Figure S1). The biological processes that stood out as most relevant to the disease state included striated muscle contraction, muscle development, cytoskeletal anchoring, negative regulation of metabolism, protein ubiquitination, and RNA processing. The cellular components of these genes were generally grouped into muscle components and ECM components, particularly the basement membrane. Twenty-eight different ontogenies were overrepresented based on the 99 down-regulated genes (Additional File 4 Table S3; Additional File 5 Figure S2). The two major functions of the down-regulated biological processes were fatty acid metabolism and transport. This corresponded with the molecular function ontologies involved in fatty acid/acyl CoA binding and also contained cadmium and copper ion binding. Cellular component categories were almost exclusively related to the mitochondria, however it was interesting that sheet forming collagen type IV of the basement membrane also was over represented using this analytical approach.

### Gene pathways related to muscle function

To understand muscle tissue adaptation to CP from a physiological perspective, we analyzed gene expression ratio patterns within muscle-specific pathways of gene products that interact in a given muscle function.

**Figure 5:**
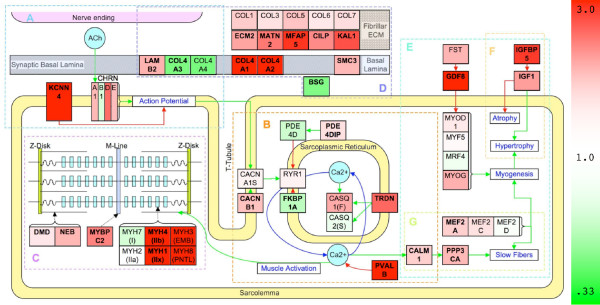
**Pathways specific to muscle analysis of transcription in CP muscle. Pathways A-G involved in muscle function**. Color is determined by the expression ratio. Up-regulated genes are red and down-regulated genes are green. Green connectors represent activation and red connectors represent inhibition in the direction of the arrow. Bolded genes represent those that are significantly altered in all three preprocessing algorithms. Italic genes (RAS, MAPK) are sets of genes involved in muscle MAPK pathway in muscle, but are not colored by expression because many individual genes are applicable and not altered in CP. Pathways represented are A: NMJ, B: ECC, C: MC, D: ECM, E: MYG, F: FT, and G: IGF1.

Because CP is a neurological disorder, a pathway describing the neuromuscular junction (NMJ) was created (NMJ; Figure 5A). No postsynaptic genes were significantly altered in CP including subunits of the nACHR receptor. Collagen type IV subunits (*COL4A3*; 2.89 and *COL4A4*; 3.26) and laminin (*LAMB2*; 1.74) of the synaptic basal lamina were significantly up-regulated. A Ca^2+^-activated K^+ ^channel (*KCNN3*; 12.98), was dramatically up-regulated in CP.

The process of converting the action potential into muscle contraction is referred to as excitation-contraction coupling (ECC; Figure 5B). The β1 regulatory subunit of the L-type voltage gated Ca^2+ ^channel was significantly up-regulated in CP (*CACNB1*; 1.59). Although the ryanodine receptor responsible for releasing Ca^2+ ^from the sarcoplasmic reticulum (SR) was not altered, the genes *FKBP1A *(0.56) and *PDE4D *(0.68) that prevent channel leaking, were significantly down-regulated [35]. Myomegalin (*PDE4DIP*; 2.10) was altered significantly in CP and is thought to anchor *PDE4D *near the SR [36]. Calmodulin (*CALM1*; 1.70) was significantly up-regulated. The most drastic change with CP on gene expression was in the up-regulation of muscle-relaxing protein, parvalbumin (*PVALB*; 62.6). The process of pumping Ca^2+ ^back into the SR is assisted by triadin (*TRDN*; 2.39), which was significantly up-regulated.

Muscle contraction obviously requires the myosin heavy chain motor and a cytoskeletal framework (MC; Figure 5C). *MYH1 *(type 2X MHC; 8.57) was significantly up-regulated in CP along with *MYH4 *(type 2B MHC; 4.33) a gene not normally expressed in humans [37]. The developmental MHCs, *MYH3 *(embryonic MHC; 15.74) and *MYH8 *(perinatal MHC; 7.74) showed large up-regulation. *MYH7 *(type 1 MHC; 0.72) was unchanged. The *MYBPC2 *(2.16) is a fast isoform of myosin binding protein and was up-regulated in CP. Several sarcomeric structural components were also up-regulated as well: dystrophin (*DMD*; 2.11), nebulin (*NEB*; 2.54), and muscle LIM domain binding protein 3 (*LDB3*; 1.93).

We suspected that ECM transcription would be altered based on previous biomechanical results ([11,13,38]; ECM; Figure 5D). Fibrillar collagens all increased modestly. Interestingly, basal laminar collagen IV was altered with *COL4A1 *(0.51) and *COL4A2 *(0.43) decreasing significantly while *COL4A3 *(2.89) and *COL4A4 *(3.26) increased significantly. Basigin, (*BSG*; 0.48) was significantly down-regulated. Various other ECM components were also up-regulated in CP: *ECM2 *(1.86), *KAL1 *(2.57), *MATN2 *(2.24), *MFAP5 *(3.64), *CILP *(2.09) and *SMC3 *(1.88).

Myogenesis describes the pathway that produces muscle growth (MYG; Figure 5E). *IGF1 *(2.63) was up-regulated along with *IGFBP5 *(2.48) (IGF1; Figure 5F). Myostatin (*GDF8*; 3.65), an inhibitor of myogenesis, was also significantly up-regulated. Other significantly up-regulated genes implicated in myogenesis are *NEO1 *(1.44, [39]), *PLCB1 *(2.34, [40]), *PBX1 *(1.86, [41]), and *HMGB1 *(1.65, [42]), *MBNL1 *(1.74, [43]), and MAPK6 (1.77, [44]). However, the muscle regulatory factors (*MYOD1 *1.22; *MYF6 *0.87; *MYF5 *1.06; *MYOG *1.75) did not show a significant transcriptional change. Mitogen activated protein kinases (MAPK) have been proposed as a major pathway in muscle hypertrophy [45], however our study showed minimal transcriptional affects on this signaling family. Another segment of myogenesis relates to satellite cell activation, proliferation and incorporation into adult muscle fibers. None of the markers for quiescent or activated satellite cells (quiescence: *PAX7*, *FOXK1*, *MET*, *CDH15*, *NCAM1*, *VCAM1*, *SDC3*, *SDC4*; activation: *MYF5*, *MYOD1*, *MYOG*, *MYF6*, *PCNA*, *CDKN1A*, *MYH3*, *MYH8*) were significantly altered in CP suggesting minimal involvement of satellite cells in the disease. Additional cell cycle transcripts were investigated, but did not show a significant change.

Although slow fiber creation is related to myogenesis, there is also a specific pathway for slow oxidative muscle fiber type determination (FT; Figure 5F). Sensing and signaling factors, *CALM1 *(1.70) and calcineurin (*PPP3CA*; 1.95) respectively, had significantly increased transcription along with transcription factor *MEF2A *(1.53), but NFATs and other MEF2 expressions levels were unchanged.

### Cerebral palsy compared to other muscle pathologies

To determine whether the CP transcriptome was unique or simply a secondary adaptation of decreased activity in these children (as might be observed with immobilization (IMB)), or whether the response was a generic muscle pathology (Duchenne Muscular Dystrophy (DMD) being the most-commonly studied), or was similar to spastic muscle in an alternative more developed muscle (Hereditary Spastic Paraplegia (HSP) being a spastic condition with adult subjects) we compared our GeneChip data to these three muscle pathologies for which GeneChip data were available [27-29]. To make these comparisons, the expression ratio values for the pathways were compared amongst the three conditions (Table 4). While averaging over an entire pathway may be misleading (similar scores may result from different gene expression patterns), different scores do emphasize pathways that are unique among disease states. This analysis revealed significant satellite cell activation, as expected, in DMD [27] as well as increased NMJ components (primarily nicotinic acetylcholine receptor subunits) and loss of contractile material as expected in IMB [28]. HSP represents muscle adaptation to altered neuronal input, although there was a negative correlation in most pathways, ECC seemed to be handled in a similar manner. CP was unique relative to the other two pathologies based on the IGF1 pathway increase, slow fiber activation, and increased expression of ECC activators and inhibitors. Thus, the correlation data support the assertion that CP is unique relative to other disease states.

**Table 4:** Changes in pathways correlated with other muscle disease states

	**CP**	**DMD**		**IMB**		**HSP**	
	MEAN	MEAN	CORR	MEAN	CORR	MEAN	CORR
*NMJ*	2.39	0.98	-0.24	1.19	-0.05	0.97	-0.02
*ECC*	0.66	0.90	0.12	1.26	0.14	1.39	0.62
*MC*	2.91	2.18	0.91	0.65	-0.74	1.07	-0.48
*ECM*	1.63	1.73	-0.23	0.69	0.44	0.66	0.02
*FT*	1.35	0.70	-0.21	0.92	0.37	0.91	-0.79
*IGF1*	1.19	1.58	N/A	1.04	N/A	0.76	N/A
*MYG*	1.14	1.11	-0.16	1.01	-0.24	1.01	-0.09
*SCQ*	1.19	1.18	-0.67	1.11	0.66	1.23	0.07
*SCA*	2.17	3.73	0.91	0.86	-0.81	0.94	-0.54

Quantification of gene pathways in various disease states (CP, DMD, IMB, HSP). Quantification represents MEAN (geometric mean of expression ratios in specific muscle pathways defined in Figure 5 using inverse values for pathway inhibitors) and CORR (correlation of CP with the other disease states, reported with an R value). Satellite cells markers are separated into genes expressed in the quiescent (SCQ) and activated states (SCA).

## Discussion

The purpose of this study was to define the muscle transcriptional adaptations in children with cerebral palsy (CP) to gain insights into the cellular mechanisms that might explain muscular adaptation in this neurological condition. We show that the transcriptional profile of CP muscle is fundamentally different compared to normal controls (Figure 1). Previous CP muscle studies of intraoperative sarcomere length [12], in vitro tissue biomechanics [38,46], and immunohistochemical and biochemical assays suggested adaptation of extracellular matrix regulation [11,13], myogenenic pathways [10,38], and fiber type determination pathways [25] in this condition. Our transcriptional analyses provide potential explanations of the cellular bases for these adaptations. Based on a general understanding of muscle physiology and biology, we placed the gene expression patterns into the context of six major muscle physiological systems – the neuromuscular junction (Figure 5A), excitation-contraction coupling (Figure 5B), muscle contraction (Figure 5C), extracellular matrix regulation (Figure 5D), myogenesis (Figure 5E) and fiber type determination (Figure 5F). As will be seen, one feature of CP is that conflicting tendencies occur within and between these various systems.

The initial insult in CP is located in the central nervous system, but this primary insult leads to a secondary effect on the skeletal muscle system. Thus the NMJ, as the nerve-muscle interface, may play a role in CP. Studies have shown disrupted NMJ in that acetylcholine receptors appear outside the NMJ area more often in CP, although they were unable to find any change in transcriptional regulation [47,48]. However, *KCNN3 *was the 2^nd ^most up-regulated gene on the entire chip and this gene plays a role in causing after-hyperpolarizations which may be a cellular attempt to limit the excessive motor unit firing that has been reported in spastic muscle [49]. Interestingly, *KCNN3 *is usually expressed in immature muscle and inhibited after innervation, which may indicate a sort of "immature state" of this muscle [50]. Although their localization in these samples is unknown the standard collagenous component of the synaptic basal lamina (*COL4A3*; *COL4A4*) was transcriptionally increased, the opposite activity of primary muscle basal lamina collagens (*COL4A1*; *COL4A2*) [51]. If these synaptic collagen IV subunits occurred outside the NMJ it would suggest a further degree of NMJ disorganization, alternatively they could be another indicator of muscle in an "immature state."

We also uncovered significant evidence of altered calcium handling secondary to CP. Our data appear to reflect chronically increased intracellular calcium since the L-type voltage gated Ca^2+ ^channel (*CACNB1*) was up-regulated (leading to activation of the ryanodine receptor) and leakage through the ryanodine receptor would be increased by down-regulation of two genes that prevent leakage (*FKBP1A*; *PDE4D*). Another "attempt" by the muscle to re-regulate [Ca]_i _can be inferred by the up-regulation of *TRDN*, which reclaims Ca^2+ ^to the SR by localizing calsequestrin within the SR [52]. Chronically altered calcium levels and subsequent activation of the intramuscular calcium-activated proteases (Calpains) would cause dramatic muscle lesions, although they are not transcriptionally regulated in CP. Indeed, a relatively new class of Calpain-mediated myopathies has recently been described [53,54]. Perhaps in response to this chronic change in [Ca]_i _a huge 63-fold increase in *PVALB*, a Ca^2+ ^binding protein was induced in order to force muscle relaxation [55]. This dramatic adaptation could have significant effects on the [Ca]_I _and may even lower it below control levels and alter muscle contractile properties.

Of the proteins involved in calcium induced force generation, MHC isoforms are the most responsive to CP. They are primarily responsible for determining muscle fibers type [56] and undergo a transformation in the direction of a slow-to-fast phenotype. This shift included immature myosins, which saw large increases, although they were only significant in 2/3 algorithms, and lends further evidence to muscle in an "immature state." The many oxidative metabolic genes that are down-regulated in CP (Table 3) support this slow-to-fast transition. The ontology analysis revealed the loss of metabolic and mitochondrial related transcripts represented the majority of down regulated ontologies (Additional File 4 Table S3). Although previous research is mixed on whether spastic muscles become more fast or slow, our data is in concordance with recent research that fast fibers dominate spastic muscle in CP [25,34]. Paradoxically, this transformation occurs despite an overall increase in gene transcription related to the determination of the slow fiber phenotype, particularly calmodulin (*CALM1*) and calcineurin (*PPP3CA*) [57]. A potential explanation may be that the dramatic PVALB expression actually leads to a decrease in intracellular calcium, thus turning off the initiation of the slow gene program. The validity of the calcineurin/NFAT pathway for transcription of a slow muscle fiber program has also come under question [58,59].

The slow fiber program represents only one segment of myogenesis that is controlled by many other genes. While the majority of the pathway elements (receptors, second messengers, signaling molecules) involved in myogenesis were not changed, two of the most important initial factors were both up-regulated – insulin-like growth factor (*IGF1*) and myostatin (*GDF8*). Interestingly they produce opposing effects on myogenesis with IGF1 leading to hypertrophy and myostatin opposing growth [60,61]. What this means for the net level of myogenesis is unclear. Satellite cells are an important contributor to muscle growth, but their role in CP is difficult for us to discern as neither quiescent nor activated satellite cell markers were altered transcriptionally.

Muscle development was indicated in the ontology analysis (Additional File 2 Table S2) and some genes related to myogenesis were up-regulated (Table 3). While the muscle regulatory factors were not significantly altered, apparently fewer "growth" proteins must be activated since muscle growth in children with CP is decreased [10]. The reduction of parallel growth would lead to decreased muscle strength in CP patients. Reduced longitudinal growth would limit range of motion, and this has been suggested as the cause for extraordinarily long in vivo sarcomere lengths in children with wrist flexion contractures [12]. The increase in *GDF8 *could be responsible for this lack of growth in spastic CP muscle and thus represent a potential therapeutic target. Other evidence pointing toward muscle degradation is in the expression ontology of protein ubiquitination being increased, based on the up-regulation of 4 related genes (*FBXO3*, *PCNP*, *RBBP6*, and *UBE2V2*) and supported by an up-regulation of *CACYBP*, a gene involved in calcium dependent ubiquitination. The opposing actions of IGF1 to increase muscle mass are also controlled by a number of IGF binding proteins and we revealed *IGFBP5 *was significantly up-regulated in CP, however the effects of *IGFBP5 *in muscle have been questioned [62,63]. These results make the activation of the IGF1 pathway difficult to decipher at the transcriptional level. Furthermore, the hypertrophic effect of IGF1 is primarily from an increase in translation efficiency, which could have broad effects but would be unobservable in our study.

One of IGF1's broad anabolic effects could be a contribution to the increased ECM in muscle from CP patients [64,65]. While the ECM is altered transcriptionally, it is unclear which components are most affected. The fibrillar components of collagen in muscle are primarily collagen types I and III and each alpha chain of these collagen types were slightly up-regulated. The most dramatic changes were in the collagens of the basal lamina discussed in reference to the NMJ. Overall the basal lamina has been demonstrated as an area of excessive growth, and thus may be important in understanding muscle pathology [13]. Gene ontology analysis revealed a set of genes associated with the ECM that were all significantly up-regulated (Additional File 2 Table S2). This supports the hypothesis of a prolific ECM in spastic muscle of CP patients. The decreased transcription of basigin (*BSG*; 0.48) could also lead to extensive ECM through the reduced activation of MMPs [66]. Basigin may also implicate a disorganized ECM lacking full functionality as MMP activity is usually increased along with increases in ECM production. However TIMP's are the primary MMP inhibitors and did not show a corresponding transcriptional increase [67].

It is important to note the distinct pathology of CP, as spastic muscle does not fit neatly into any of the other "altered use" muscle models [68]. The transcriptional control of muscle in CP was qualitatively different compared to DMD, IMB, or HSP (Table 4). DMD actually showed the most similarity to CP, particularly among contractile genes and satellite cell markers. DMD is known to have activated satellite cells and regenerating muscle and this correlation implicates the same in CP, although no satellite cell activation markers were significantly up-regulated in CP. IMB and HSP had an opposing effect on satellite cells, which shows this is not consistent with all disease states. IMB has been shown to result in muscle atrophy, fibrosis, and a shift from slow to fast muscle fibers. Although we were not able to compare our results to a human overactivity microarray study we clearly did not show the increase in slow fibers and mitochondrial transcripts expected. In fact HSP, which may be expected to the most similar to CP, resulted in little correlation (R < 0.1) with CP in all pathways except ECC. This suggests that ECC alterations may be a defining characteristic of spastic muscle. It is also interesting to note that, in the other cases, *IGF1 *and *GDF8 *acted alternatively – IGF1 increased while GDF8 decreased in DMD and conversely for IMB and HSP. This highlights the unique adaptation of CP, where myogenesis is turned on and off simultaneously.

While we are able to demonstrate the transcriptional effects of CP we also investigated this effect on two separate muscles and at different levels of clinical severity. Tendon transfer surgery is relatively common procedure for CP patients and is implicated when there is a muscular imbalance around a joint. It involves transferring the distal tendon of a muscle on the side of a joint considered to have a contracture or relative over activity to a tendon on the opposing side of the joint. Transfer of FCU to ECRB to correct wrist position is one of the common tendon transfer surgeries. Thus FCU is considered the more pathologic muscle and we might have expected a different transcriptional profile. However, we were unable to show any transcriptional differences between the muscles, indicating that both wrist flexors and extensors have a similar adaptation to CP. While the FCU is known to exhibit contractures in CP, we conclude that the contracture is developed due to its architecture, not due to a fundamental difference is secondary adaptation to the altered neuronal input of CP. The FCU is a larger muscle than the ECRB and the larger wrist flexor muscles may simply dominate the disease state based on their size. We were also unable to show significant transcriptional differences among various clinical severity scores in CP patients. This may be because CP transcriptional profiles are either on or off. More likely our study was unable to resolve a severity effect as the study is biased towards the most severe cases (patients recruited based on corrective surgery) or the study is simply underpowered. We would likely need more patients across the range of clinical severity scores to define the genes most closely correlated with severity. However the low power of the severity analysis is increased in our comparisons of CP vs. control muscle. Further, a discussion of statistical power does not apply to significant differences detected in CP vs. control muscle. We do acknowledge, however, that we are clearly not detecting all transcripts that are altered in CP.

Our study has some inherent limitations, one of which is the small sample size noted above, especially in the case of control patients. As with any human study there is a high degree of heterogeneity among the samples. These patients have been treated in a variety of ways, and it is important to note that our transcriptional profile is not solely based on CP, but includes conservative treatment. We must also point out that this muscle is in a chronic disease state, making it difficult to discern the primary effects of CP from compensatory mechanisms that have taken place. As with any GeneChip study, we discuss only transcriptional control and any observation is subject to post-transcriptional modification.

Despite these inherent limitations we have been able to highlight areas where future work on spastic CP muscle may lead to innovative therapies. Our altered calcium handling data points to chronically elevated calcium levels which are highly dangerous since they may activate endogenous proteases. Fortunately a variety of calcium channel blockers have been developed and tested which could be of use in treating CP. Another potential application of current techniques could come from antifibrotic therapy to combat the increase in ECM components which is suggested by the transcriptomes. Of the most promising may be myostatin inhibiters, currently under investigation, since growth is inhibited in muscle from CP patients and myostatin, a major inhibiter of muscle growth is significantly up-regulated. This transcriptional study helps point the way to these and other areas of protein modifications, cell signaling, and biomechanics where future investigations should be focused.

## Conclusion

Dramatic transcriptional alterations occur in muscle secondary to CP. These transcriptional changes ultimately lead to derangement of the ECM components of spastic muscle along with alteration of transcripts involved in myogenesis. A number of genes alter their expression in order to create a slow-to-fast transition of MHC isoforms and metabolic profile. GeneChip analysis has also allowed us to demonstrate the many changes in Ca^2+ ^handling occur in CP that was not suggested previously. Together we are able to postulate the mechanisms known to affect muscle function in CP and predict new ones. This will aid future research into CP muscle and therapies to treat CP patients.

## Abbreviations

(CP): Cerebral palsy; (ECC): excitation contraction coupling pathway; (ECM): extra-cellular matrix pathway; (ECRB): extensor carpi radialis brevis; (DMD): Duchenne muscular dystrophy; (FCU): flexor carpi ulnaris; (FT): fiber type pathway; (GCRMA): GC robust multichip analysis; (GEO): Gene Expression Omnibus; (HSP): hereditary spastic paraplegia; (IGF1): IGF1 pathway; (IMB): immobilization; (LMN): lower motor neuron; (MAS5): microarray suite version 5.0; (MC): muscle contraction pathway; (MYG): myogenesis pathway; (NMJ): neuromuscular junction pathway; (QPCR): quantitative polymerase chain reaction; (RMA): robust multichip analysis; (SCA): satellite cell activation markers; (SCQ): satellite cell quiescence markers; (SR): sarcoplasmic reticulum; (UMN): upper motor neuron.

## Competing interests

The authors declare that they have no competing interests.

## Authors' contributions

LRS carried out the RNA isolation, qPCR experiments, genechip analysis, and drafted the manuscript. EP provided the biopsies and assisted in review of the manuscript. YH carried out the myosin heavy chain content experiments. SRW participated in critical review of the manuscript. HC provided expertise on CP and critical review of the manuscript. SS provided expertise on genechip analysis and critical review of the manuscript. RLL conceived of the study, and participated in its design and coordination and supervised the writing of the manuscript. All authors have read and approve of this manuscript.

## Pre-publication history

The pre-publication history for this paper can be accessed here:



## Supplementary Material

Additional file 1 **Significantly altered genes in CP**. List of all features with a significant p-value (< 0.05) using each preprocessing algorithm (MAS5, RMA, GCRMA). Ratio of CP/CTRL is determined using MAS5. Click here for file

Additional file 2 **Significantly up-regulated Gene Ontologies in CP**. List of all Gene Ontologies significantly up-regulated genes in CP. O: observed genes, E: expected genes, R: ratio of observed/expected, P: p-value. Click here for file

Additional file 3 **Significantly up-regulated Gene Ontologies tree in CP**. Hierarchical list of Gene Ontologies in CP with red lettering representing significantly up-regulated Gene Ontologies. Click here for file

Additional file 4 **Significantly down-regulated Gene Ontologies in CP**. List of all Gene Ontologies significantly down-regulated genes in CP. O: observed genes, E: expected genes, R: ratio of observed/expected, P: p-value. Click here for file

Additional file 5 **Significantly down-regulated Gene Ontologies in CP**. Hierarchical list of Gene Ontologies in CP with red lettering representing significantly down-regulated Gene Ontologies. Click here for file
