# Supplementary figures and images for: Novel transcriptional profile in wrist muscles from cerebral palsy patients
Source: BMC Med Genomics. 2009 Jul 14;2:44. doi: 10.1186/1755-8794-2-44 (PMC2722667; doi:10.1186/1755-8794-2-44)

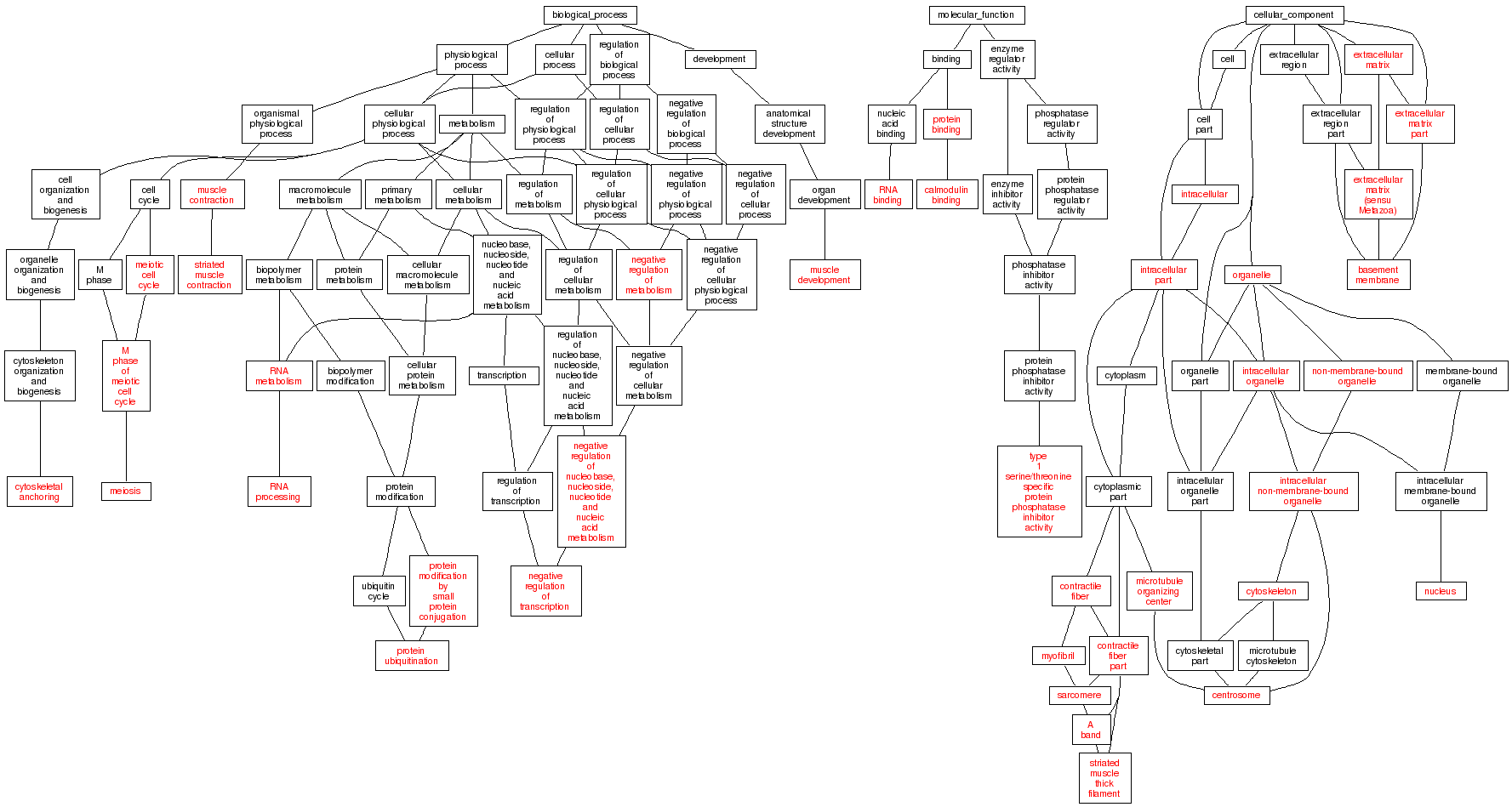

Supplement: Additional file 3 — Significantly up-regulated Gene Ontologies tree in CP. Hierarchical list of Gene Ontologies in CP with red lettering representing significantly up-regulated Gene Ontologies. [file 1755-8794-2-44-S3.gif]

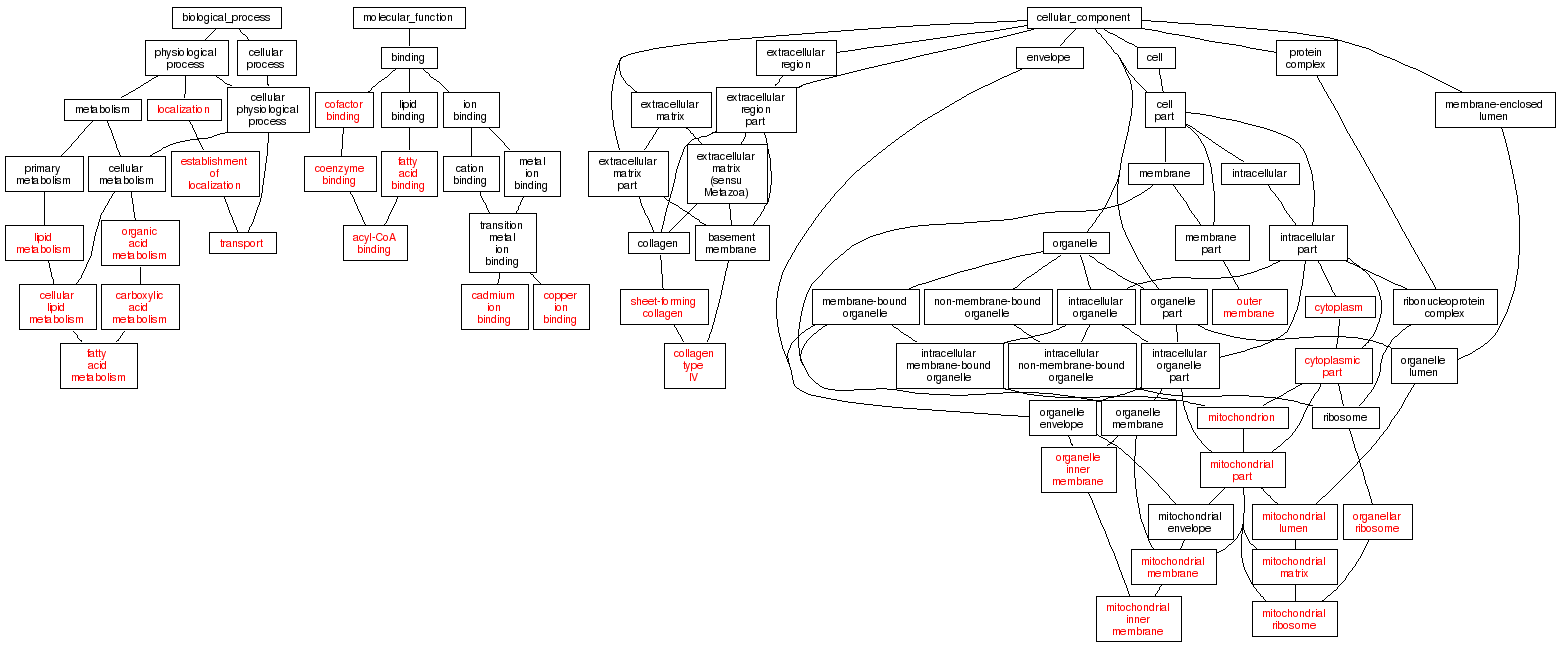

Supplement: Additional file 5 — Significantly down-regulated Gene Ontologies in CP. Hierarchical list of Gene Ontologies in CP with red lettering representing significantly down-regulated Gene Ontologies. [file 1755-8794-2-44-S5.gif]
